# Supplementary figures and images for: An in silico molecular docking and simulation study to identify potential anticancer phytochemicals targeting the RAS signaling pathway
Source: PLoS One. 2024 Sep 19;19(9):e0310637. doi: 10.1371/journal.pone.0310637 (PMC11412525; doi:10.1371/journal.pone.0310637)

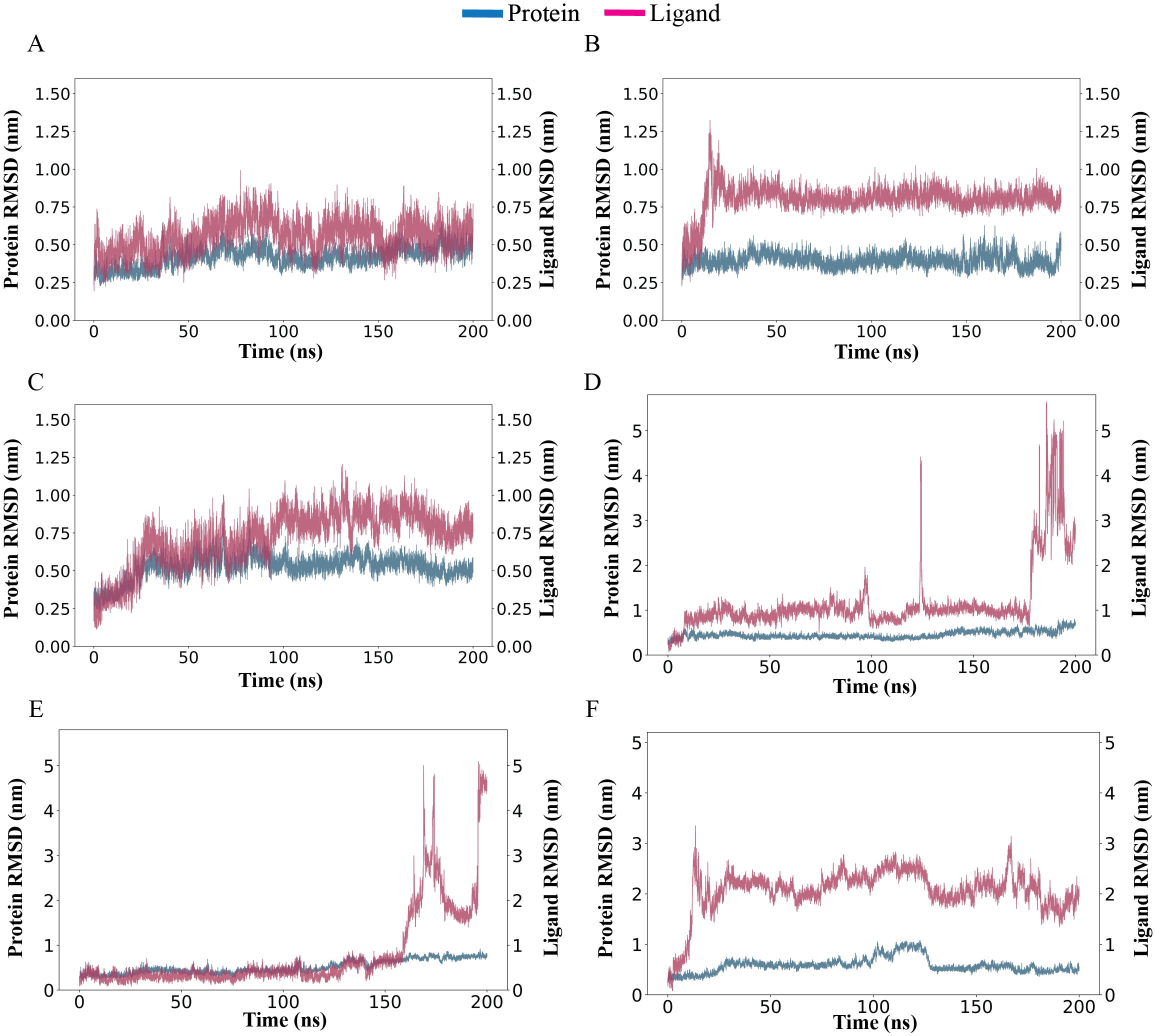

Supplement: S1 Fig — A) RMSD of Complex 1 B) RMSD of Complex 2, C) RMSD of Complex 4, D) RMSD of Complex 5, E) RMSD of Complex 6, and F) RMSD of Complex 9. The root means square deviation (RMSD) between the ligand and protein exhibits temporal inconstancy, thereby ensuring instability. Complex 1 showed stability for a certain time and then the ligand was out of the protein contact at 75 to 100ns, indicating poor stability. (TIF) [file pone.0310637.s001.tif]

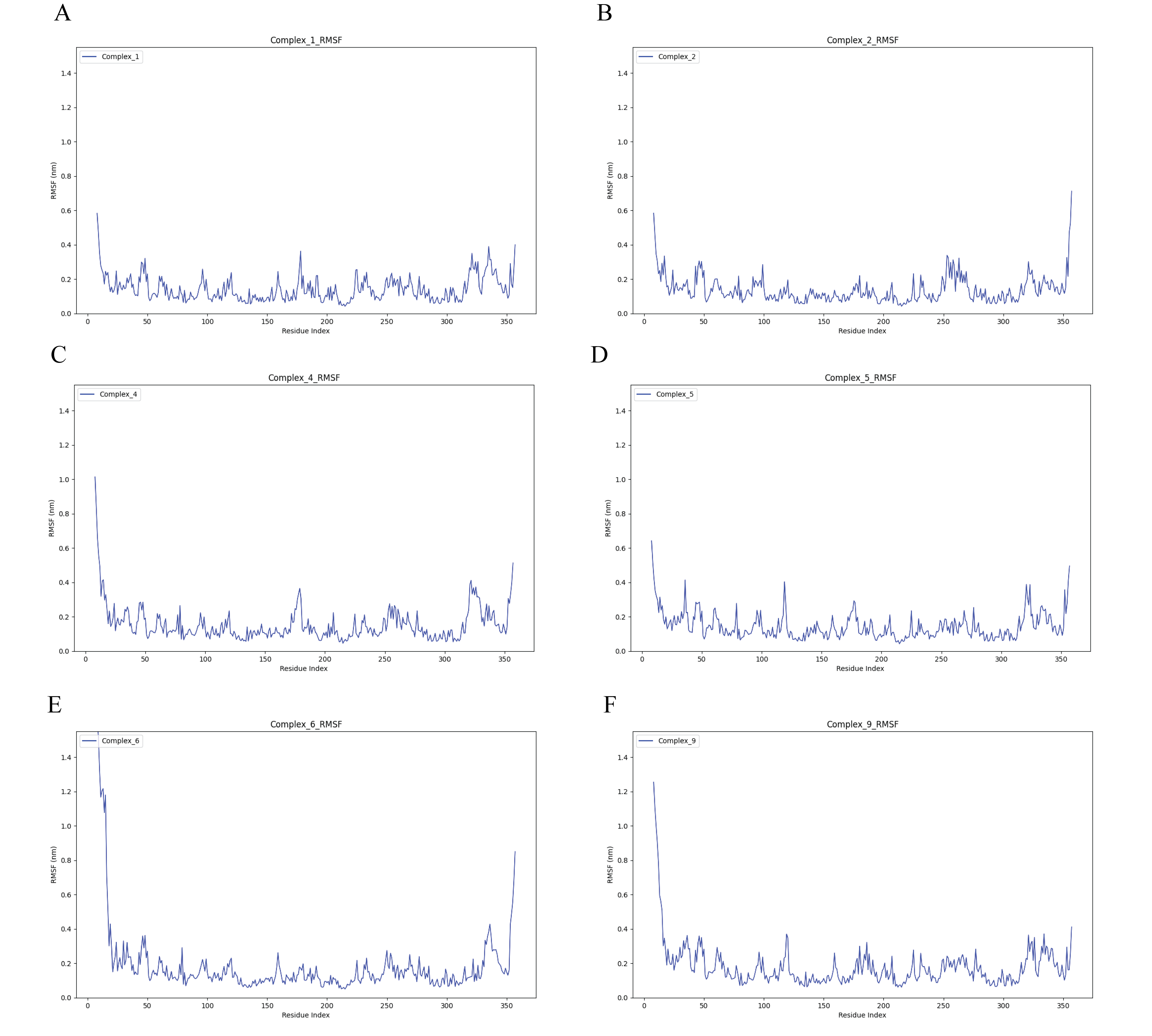

Supplement: S2 Fig — A) RMSF of Complex 1 B) RMSF of Complex 2, C) RMSF of Complex 4, D) RMSF of Complex 5, E) RMSF of Complex 6, and F) RMSF of Complex 9. The interpretation of the results is justified by Several significant fluctuations. The fluctuation primarily arises when the ligand interacts with the protein residues. All the complexes exhibit several significant fluctuations not more than 0.4nm. (TIF) [file pone.0310637.s002.tif]

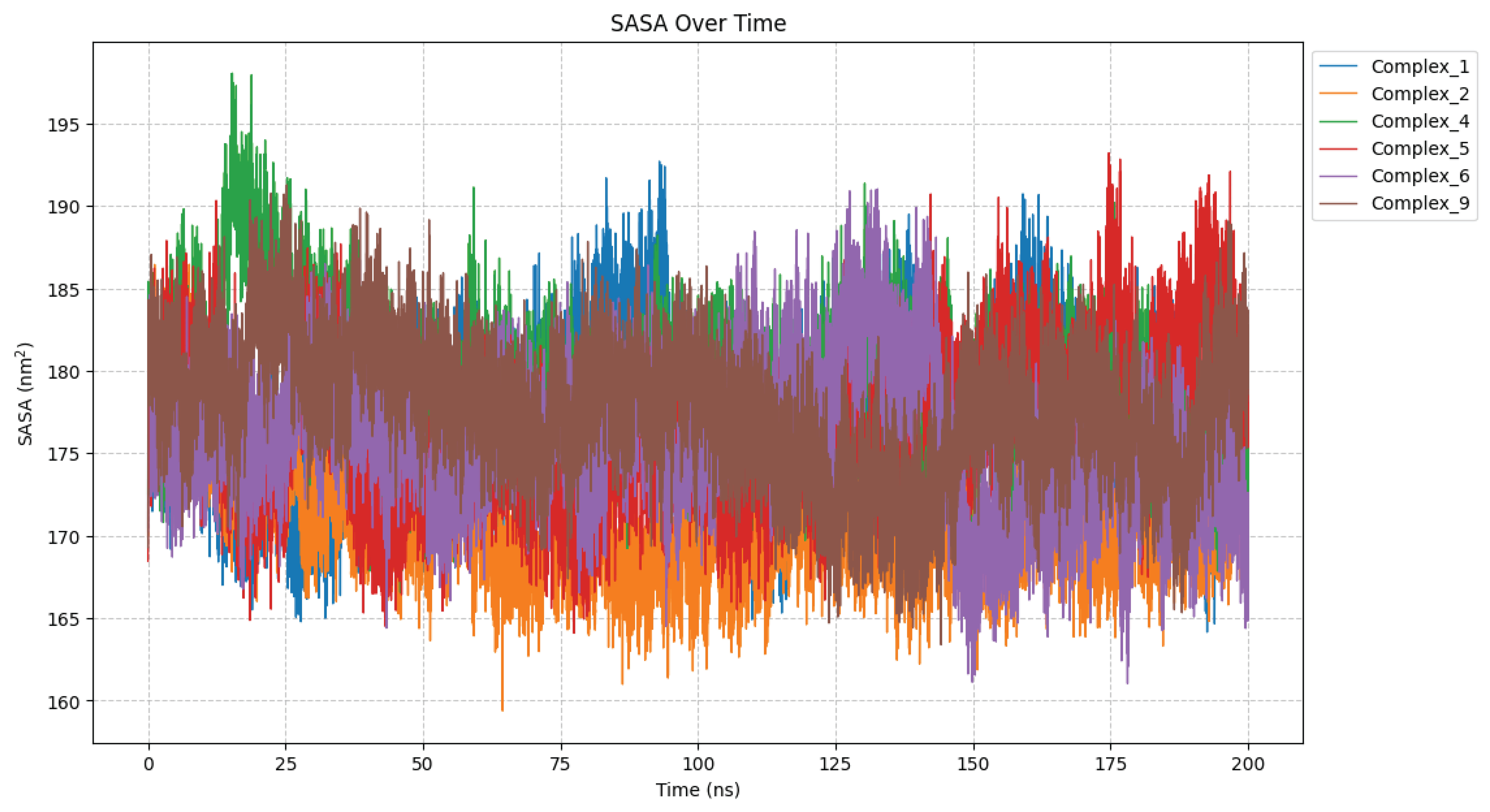

Supplement: S3 Fig — Protein and the control SASA are displayed in main Fig 3. The SASA calculation was between 160 to 195 nm2. The solvent-accessible surface area (SASA) measurements of the amino acid residues at the C-terminus of one protein are found to be lower than those at the N-terminus of a different protein, indicating a higher degree of hydrophobicity and compactness in the free end amino acid residues of the former protein in comparison to the latter. (TIF) [file pone.0310637.s003.tif]
