# Supplementary material for: An in silico molecular docking and simulation study to identify potential anticancer phytochemicals targeting the RAS signaling pathway
Source: PLoS One. 2024 Sep 19;19(9):e0310637. doi: 10.1371/journal.pone.0310637 (PMC11412525; doi:10.1371/journal.pone.0310637)
Supplement: S1 Table — (PDF) [file pone.0310637.s005.pdf]

S1 Table. The Active side residues

**Sequence ID**	**31**	**32**	**33**	**34**	**36**	**37**	**39**	**52**	**53**	**54**	**56**	**64**
Amino acid	ILE	GLY	GLU	GLY	TYR	GLY	VAL	ALA	ILE	LYS	ILE	TYR
**Sequence ID**	**67**	**68**	**71**	**75**	**84**	**103**	**105**	**106**	**107**	**108**	**109**	**110**
Amino acid	ARG	THR	GLU	LEU	ILE	ILE	GLN	ASP	LEU	MET	GLU	THR
**Sequence ID**	**111**	**113**	**114**	**149**	**151**	**153**	**154**	**156**	**166**	**167**	**169**	
Amino acid	ASP	TYR	LYS	ASP	LYS	SER	ASN	LEU	CYS	ASP	GLY	
