# Supplementary material for: An in silico molecular docking and simulation study to identify potential anticancer phytochemicals targeting the RAS signaling pathway
Source: PLoS One. 2024 Sep 19;19(9):e0310637. doi: 10.1371/journal.pone.0310637 (PMC11412525; doi:10.1371/journal.pone.0310637)
Supplement: S2 Table — (PDF) [file pone.0310637.s006.pdf]

S2 Table. ADMET Profiling of Sorted Compounds

Chemical	CID	MW	Heavy atoms	Rotatable bonds	H-bond acceptors	H-bond donors	Max. tolerated dose (human)	Skin Sensitisation	Minnow toxicity
Erk_inhibitor	135523966	459.3	31	7	4	4	0.716	No	1.718
(+)-Catechin	9064	290.27	21	1	6	5	0.438	No	3.585
Apigenin	5280443	270.24	20	1	5	3	0.328	No	2.432
Aromadendrin	122850	288.25	21	1	6	4	0.353	No	3.754
Axillarin	5281603	346.29	25	3	8	4	0.506	No	2.979
Chrysoeriol	5280666	300.26	22	2	6	3	0.436	No	1.654
Cirsilineol	162464	344.32	25	4	7	2	0.275	No	1.706
Citrinin	54680783	250.25	18	1	5	2	1.372	No	2.046
CURCUMIN	969516	368.38	27	8	6	2	0.081	No	-0.081
Diosmetin	5281612	300.26	22	2	6	3	0.42	No	1.737
Epicatechin	72276	290.27	21	1	6	5	0.438	No	3.585
Eriodictyol	440735	288.25	21	1	6	4	0.014	No	2.972
Eupatorin	97214	344.32	25	4	7	2	0.262	No	1.691
Galangin	5281616	270.24	20	1	5	3	0.333	No	2.385
Genistein	5280961	270.24	20	1	5	3	0.478	No	1.941
Hesperetin	72281	270.24	22	2	6	3	0.25	No	2.305
Hispidulin	5281628	302.28	22	2	6	3	0.279	No	1.64
Isorhamnetin	5281654	300.26	23	2	7	4	0.576	No	2.206
Kaempferol	5280863	316.26	21	1	6	4	0.531	No	2.885
Luteolin	5280445	286.24	21	1	6	4	0.499	No	3.169
Melodorinol	5388649	286.24	19	5	5	1	0.663	No	1.622
Pelargonidin	67249	260.24	21	1	5	4	0.491	No	2.35
Quercetin	5280343	306.7	22	1	7	5	0.499	No	3.721
Rhamnetin	5281691	302.24	23	2	7	4	0.56	No	1.885
Rhein	10168	316.26	21	1	6	3	0.716	No	2.547
Scutellarein	5281697	284.22	21	1	6	4	0.626	No	1.99
Taxifolin	439533	286.24	22	1	7	5	0.345	No	4.688
