# Supplementary material for: An in silico molecular docking and simulation study to identify potential anticancer phytochemicals targeting the RAS signaling pathway
Source: PLoS One. 2024 Sep 19;19(9):e0310637. doi: 10.1371/journal.pone.0310637 (PMC11412525; doi:10.1371/journal.pone.0310637)
Supplement: S3 Table — (PDF) [file pone.0310637.s007.pdf]

S3 Table. Triplet docking scores (autodock vina and pyrx) with an average score in Kcal/mol unit.

Chemical	CID	Autodock Vina 1	Autodock Vina 2	Autodock Vina 3	Average (Autodock Vina)	Pyrx 1	Pyrx 2	Pyrx 3	Average (Pyrx)
Erk_Inhibitor	135523966	-9.5	-10.1	-9.1	-9.56	-8.5	-8.5	-8.7	-8.56
Chrysoeriol	5280666	-10.2	-10.2	-10.2	-10.2	-8.5	-8.3	-8.3	-8.36
Luteolin	5280445	-10.1	-10.1	-10.1	-10.1	-8.4	-8.7	-8.7	-8.6
Quercetin	5280343	-10.1	-10.1	-10.1	-10.1	-8.6	-8.4	-8.4	-8.46
Rhamnetin	5281691	-9.9	-10.1	-10.0	-10	-8.6	-8.4	-8.4	-8.46
Hispidulin	5281628	-9.9	-9.9	-9.8	-9.86	-8.4	-8.4	-8.4	-8.4
Apigenin	5280443	-9.7	-9.8	-9.9	-9.8	-8.2	-8.6	-8.6	-8.46
Isorhamnetin	5281654	-9.8	-9.8	-9.7	-9.76	-8.2	-8.2	-8.2	-8.2
Genistein	5280961	-9.9	-9.8	-9.7	-9.8	-8.7	-8.5	-8.8	-8.66
Kaempferol	5280863	-9.7	-9.7	-9.7	-9.7	-8.1	-8.1	-8.1	-8.1
Scutellarein	5281697	-9.1	-10.1	-9.5	-9.56	-8.5	-8.6	-8.6	-8.56
Cirsilineol	162464	-9.5	-9.5	-9.5	-9.5	-7.8	-7.8	-7.7	-7.76
Pelargonidin	67249	-9.2	-9.6	-9.6	-9.46	-7.9	-8.0	-7.6	-7.83
Taxifolin	439533	-9.4	-9.4	-9.4	-9.4	-8.3	-8.3	-8.3	-8.3
Aromadendrin	122850	-9.3	-9.3	-9.3	-9.3	-8	-8.1	-8.1	-8.06
Epicatechin	72276	-9.3	-9.3	-9.3	-9.3	-8.1	-8.4	-8.4	-8.3
Eriodictyol	440735	-9.2	-9.2	-9.2	-9.2	-8.2	-8.1	-8.0	-8.1
Axillarin	5281603	-9.1	-9.2	-9.2	-9.16	-8.3	-7.7	-7.7	-7.9
Galangin	5281616	-9.1	-9.2	-9.2	-9.16	-8.5	-8.5	-8.5	-8.5
Rhein	10168	-9.2	-8.7	-8.7	-8.86	-8.1	-8.1	-8.2	-8.13
Eupatorin	97214	-8.8	-8.8	-8.8	-8.8	-7.3	-7.4	-7.4	-7.36
(+)-Catechin	9064	-8	-8.5	-8.5	-8.33	-8	-8.5	-8	-8.16
Hesperetin	72281	-8.2	-8.2	-8.2	-8.2	-8.3	-8.3	-8.3	-8.3
CURCUMIN	969516	-8	-8.1	-8.4	-8.16	-7.6	-7.7	-7.7	-7.66
Diosmetin	5281612	-7.6	-8.1	-8.8	-8.16	-8.1	-8.1	-8.1	-8.1
Melodorinol	5388649	-8	-8	-8	-8	-6.7	-7.3	-7	-7
Citrinin	54680783	-7.3	-7.5	-7.5	-7.43	-6.4	-7	-7	-6.8
