# Supplementary material for: An in silico molecular docking and simulation study to identify potential anticancer phytochemicals targeting the RAS signaling pathway
Source: PLoS One. 2024 Sep 19;19(9):e0310637. doi: 10.1371/journal.pone.0310637 (PMC11412525; doi:10.1371/journal.pone.0310637)
Supplement: Attachment — Submitted filename: Response letter.docx [file pone.0310637.s011.docx]

**Response letter**

**Response to Editor Comments: (Journal Requirements)**

1. Please ensure that your manuscript meets PLOS ONE's style requirements, including those for file naming.

**Our responses:** Thank you for your guidance. We have ensured that our manuscript now meets PLOS ONE's style requirements, including the proper file naming conventions**.**

2. We suggest you thoroughly copyedit your manuscript for language usage, spelling, and grammar.

**Our responses:** We appreciate your suggestion. We have thoroughly copyedited our manuscript for language usage, spelling, and grammar to ensure it meets the high standards of PLOS ONE.

3. Please note that PLOS ONE has specific guidelines on code sharing for submissions in which author-generated code underpins the findings in the manuscript. In these cases, we expect all author-generated code to be made available without restrictions upon publication of the work. Please review our guidelines at https://journals.plos.org/plosone/s/materials-and-software-sharing#loc-sharing-code and ensure that your code is shared in a way that follows best practice and facilitates reproducibility and reuse.

**Our responses:** We appreciate your concern. We didn’t not use any code for the present research. All the methods and procedure have been described in the methodology section properly.

4. Thank you for stating the following financial disclosure: [AG has received partial funding from the Shahjalal University of Science and Technology Research Center (LS/2023/1/01).]. Please state what role the funders took in the study. If the funders had no role, please state: "The funders had no role in study design, data collection and analysis, decision to publish, or preparation of the manuscript." If this statement is not correct you must amend it as needed. Please include this amended Role of Funder statement in your cover letter; we will change the online submission form on your behalf.

**Our Responses:** We appreciate your concern. The funders had no role in study design, data collection and analysis, decision to publish, or preparation of the manuscript. We have incorporated the statement in the cover letter.

5. We note that Figures 1, 2, and 3 in your submission contain [map/satellite] images which may be copyrighted. All PLOS content is published under the Creative Commons Attribution License (CC BY 4.0), which means that the manuscript, images, and Supporting Information files will be freely available online, and any third party is permitted to access, download, copy, distribute, and use these materials in any way, even commercially, with proper attribution. For these reasons, we cannot publish previously copyrighted maps or satellite images created using proprietary data, such as Google software (Google Maps, Street View, and Earth). For more information, see our copyright guidelines: <http://journals.plos.org/plosone/s/licenses-and-copyright>.

**Our responses:** Thank you for bringing this to our attention. We appreciate the importance of adhering to copyright guidelines. We would like to clarify that the images in Figures 1, 2, and 3 of our submission are original and not derived from copyrighted sources such as Google Maps, Street View, or Earth. The images were created using publicly available data and tools that allow for redistribution under the Creative Commons Attribution License (CC BY 4.0). Although Figure 1 needs to change for solving the reviewer question. **If further verification is required, we are more than willing to provide additional details regarding the sources and methods used to generate these images.** Thank you for your understanding and support. We look forward to your guidance on any further steps needed to ensure compliance.

**Reviewer #1:**

**Query 1.** The description in the ligand selection section lacks specifics. It appears to be more generalized than a proper methodology section.

**Our response:** Thank you for your observation. We acknowledge that the description in the ligand selection section may come across as generalized. To enhance clarity and provide a more precise methodology, we will revise this section to include specific details regarding the criteria used for ligand selection, the search parameters applied within PubChem, and the rationale for the inclusion or exclusion of certain compounds. This will ensure that the methodology is transparent and can be readily replicated by other researchers. Please check the revised version of the manuscript.

**Query 2.** The authors should clearly outline the method used to obtain the initial set of 351 phytochemicals and the criteria employed for selecting the final 27 compounds from 340 after redundancy removal. The redundancy in the discussion regarding the selection process should be addressed. The sentence "Out of 340, 27 compounds were selected based on stringent ADMET profiling criteria" also need to be discuss in scientific terms

**Our response:** Thank you for your valuable feedback. We have revised the manuscript and add the Supplementary data S1 where all the phytochemicals ADMET profiling were presented. The results have been discussed further. Please check the revised version of the manuscript.

**Query 3.** The manuscript does not adequately address the rationale behind selecting 1TVO (structure of ERK2 in complex with a small molecule inhibitor) and CASTp used for site identification to be used in the virtual screening. Furthermore, if there was a comparison made between the active site features identified by the RCSB Protein Data Bank (PDB) structure (1TVO) and those identified by CASTPA, the results of this comparison should be presented and discussed.

**Our response:** Thank you for your valuable feedback. We have revised the manuscript to better explain the rationale behind selecting 1TVO, the structure of ERK2 in complex with a small molecule inhibitor, for our study. Additionally, we have now included a detailed comparison between the active site features identified by the RCSB Protein Data Bank (PDB) structure (1TVO) and those identified by CASTp. This comparison is presented in the revised manuscript, and the results are discussed to highlight similarities in the active site identification, further strengthening the basis for our approach**.**

**Query 4.** If there was a comparison made to identify commonalities or differences between the chosen ligands and previously known inhibitors, this should be explicitly stated and the results discussed.

**Our response:** Thank you for your insightful comment. In response to your query, we have now included a detailed comparison between the predicted ligands (Luteolin, Hispidulin, Isorhamnetin) and the known ERK1/2 inhibitors (Ulixertinib and Ravoxertinib) in the discussion section. This analysis highlights the common structural features essential for binding, as well as key differences in molecular flexibility and side chain composition. We believe this addition strengthens the discussion by providing a clearer understanding of how our predicted ligands may interact with ERK1/2 compared to the known inhibitors.

**Query 5.** The lack of details about ERK2 active site parameters hinders understanding of virtual ligand selection and docking simulations. Additionally, the absence of re-docking experiments with known inhibitors weakens the validation of the virtual screening protocol.

**Our response:** Thank you for your valuable feedback. We acknowledge the concern regarding the lack of detailed ERK2 active site parameters, which indeed presents challenges in accurately predicting ligand interactions during virtual ligand selection and docking simulations. To address this, we have analyzed to mitigate these limitations. We have classified the residues with each category and have marked significant amino acids for further analysis. Regarding the absence of re-docking experiments with known inhibitors, we agree that this is an important validation step. In response, we have now conducted re-docking studies using well-characterized ERK2 inhibitors. These experiments have been included in the revised manuscript. We believe these additions address the concerns raised and enhance the robustness of our study.

**Query 6.** A more significant concern is the lack of apparent validation for the virtual screening protocol, especially considering ERK2 is a well-studied target.

In the absence of prospective validation, the authors should consider retrospective studies as suggested in below paper “doi/10.1021/jm300687e” for dude selection and https://doi.org/10.1039/C8RA09318K” to gain a better understanding of the protocol's validity.

**Our response:** We appreciate the reviewer's suggestion to include retrospective validation studies to strengthen our manuscript. While our study primarily focuses on evaluating the potential of random phytochemicals with anticancer activities to inhibit ERK1/2 and verifying their stability through simulations, we acknowledge the importance of validating the virtual screening protocol.

a. Our virtual screening protocol involves both ligand-based virtual screening (LBVS) and structure-based virtual screening (SBVS), ensuring a comprehensive evaluation of potential inhibitors. The LBVS approach identifies potential inhibitors based on their similarity to known active ligands, while SBVS involves docking these ligands into the active site of ERK1/2 to predict their binding affinities.
b. We have utilized reputable databases and tools such as Pubchem for ligand selection and molecular docking software for SBVS. These tools are widely recognized for their reliability and accuracy in virtual screening studies.
c. We acknowledge the absence of a formal decoy set in our study. However, our manuscript includes retrospective validation using known active and inactive ligands for ERK1/2. This approach allows us to benchmark our screening protocol against established inhibitors and evaluate its performance in identifying true positives and minimizing false positives.
d. Our study's primary focus is on the potential of phytochemicals as ERK1/2 inhibitors. The inherent structural diversity and unique chemical properties of phytochemicals necessitate a different approach compared to traditional synthetic compounds. Retrospective validation using phytochemical datasets aligns more closely with our study's objectives and provides relevant insights into the effectiveness of our screening protocol.
e. In light of these points, we believe our current approach, which includes retrospective validation with known ERK1/2 ligands and focuses on the unique chemical space of phytochemicals, is appropriate and relevant for our study. We appreciate the reviewer’s suggestion and will consider incorporating additional validation steps in future work to further enhance the robustness of our screening protocol.
