# Supplementary material for: An in silico molecular docking and simulation study to identify potential anticancer phytochemicals targeting the RAS signaling pathway
Source: PLoS One. 2024 Sep 19;19(9):e0310637. doi: 10.1371/journal.pone.0310637 (PMC11412525; doi:10.1371/journal.pone.0310637)
Supplement: Attachment — Submitted filename: Response letter.docx [file pone.0310637.s012.docx]

**Response letter**

**Reviewer#1**

**Query 1:** Despite the authors' rebuttal and claims of addressing the concerns, significant deficiencies persist in the manuscript. The methodology section pertaining to molecular docking of phytochemicals remains inadequate. Crucially, precise details regarding the dimension of active site, number of generated poses, along with other docking parameters are absent.

**Our responses:** Thank you for your detailed and helpful remarks. In the revised manuscript, we have expanded the section detailing the molecular docking methodology including the specific dimensions of the active site, and explicitly mention the number of poses generated during docking. For example, in the manuscript, it's mentioned that "The grid box size was set to (x=126 y=122 z=70 Å) with a center at coordinates (15.416 -0.324 13.078) the energy range was set to 4 and the exhaustiveness was set to 10. Please check the revised version of the manuscript.

**Query 2:** The authors have ignored the previous recommendation to validate the virtual screening (VS) protocol in the current study. This omission is unacceptable as it compromises the reliability of the modeling studies. Without proper validation, the results are susceptible to false positives, necessitating a reassessment of the statistical matrices used for assessment.

**Our Responses:** Thank you for your valuable and insightful comments. In response, we would like to clarify that although we attempted to conduct a decoy analysis in our previous submission, it was not successful. This time, we made every effort to perform the decoy dataset analysis, making a comparative analysis with DUDE, despite challenges with the decoy finder and software link being non-functional. We emphasize that our manuscript now includes a comprehensive decoy screening experiment. This experiment was conducted to validate the specificity of our docking results. Most phytochemicals showed strong specificity, as indicated by the significant difference in binding affinity between the active compounds and their decoys. This step is crucial in confirming that the identified compounds are specific inhibitors of the ERK2 protein, thus reducing the likelihood of false positives. Please check the methodology and result section of the revised manuscript.

**Query 3:** While the revised manuscript mentions the redocking of the co-crystal ligand, quantitative data such as Root Mean Square Deviation (RMSD) values and a comparative analysis of ligand-protein interactions between co-crystal and re-docked ligand are lacking. This information is essential for evaluating the docking protocol's performance.

**Our Responses:**  Thanks for your thoughtful and constructive comments. We have performed cross-docking with the co-crystal ligand to validate the docking protocol. The RMSD value for the cross-docked pose compared to the co-crystal reference was 0.578 Å, indicating high structural similarity. We have included the RMSD values in the manuscript, along with a comparative analysis of ligand-protein interactions between the co-crystal and re-docked ligands. Additionally, a summary of the RMSD results has been provided, highlighting how similar the re-docked poses are to the co-crystal pose. Significant interactions, such as hydrogen bonds and hydrophobic interactions between the ligands and key residues, have also been emphasized.

**Query 4:** The manuscript fails to provide clear details about the ligand preparation process following retrieval from PubChem. Pre-docking ligand optimization is a critical step that requires explicit description.

**Our Responses:** Thank you for your detailed and helpful remarks. We have clarified the ligand preparation steps post-retrieval from PubChem. The manuscript now clearly outlines that energy minimization was performed using the steepest descent algorithm, and the ligands were converted into a docking-compatible format (PDBQT). We have also specified the force fields used, along with any adjustments made to the ligands, including the setting of partial charges and defining torsional degrees of freedom.

**Query 5:** The majority of the figures in the manuscript are of poor quality and difficult to interpret. To ensure clarity and facilitate data comprehension, all figures must be presented in high resolution.

**Our Responses:** We appreciate your insightful and valuable feedback. We have addressed this by improving the resolution and clarity of the figures in the manuscript. All low-quality figures have been replaced with high-resolution images and well-labeled diagrams. Specifically, the figures related to the binding pockets and interaction analysis (e.g., Fig. 4, 5, 6, 7) have been enhanced to make them easier to interpret. Please check the revised figures after downloading from the submission site.
